# M.I.N.I.-KID interviews with adolescents: a corpus-based language analysis of adolescents with depressive disorders and the possibilities of continuation using Chat GPT

**DOI:** 10.3389/fpsyt.2024.1425820

**Published:** 2024-12-19

**Authors:** Irina Jarvers, Angelika Ecker, Pia Donabauer, Katharina Kampa, Maximilian Weißenbacher, Daniel Schleicher, Stephanie Kandsperger, Romuald Brunner, Bernd Ludwig

**Affiliations:** ^1^ Department of Child and Adolescent Psychiatry and Psychotherapy, University of Regensburg, Regensburg, Germany; ^2^ Department of Information Science, University of Regensburg, Regensburg, Germany

**Keywords:** chatbot, language analysis, depressive disorders, ChatGPT, BERT

## Abstract

**Background:**

Up to 13% of adolescents suffer from depressive disorders. Despite the high psychological burden, adolescents rarely decide to contact child and adolescent psychiatric services. To provide a low-barrier alternative, our long-term goal is to develop a chatbot for early identification of depressive symptoms. To test feasibility, we followed a two-step procedure, a) collection and linguistic analysis of psychiatric interviews with healthy adolescents and adolescents with depressive disorders and training of classifiers for detection of disorders from their answers in interviews, and b) generation of additional adolescent utterances via Chat GPT to improve the previously created model.

**Methods:**

For step a), we collected standardized interviews with 53 adolescents, *n* = 40 with and *n* = 13 without depressive disorders. The transcribed interviews comprised 4,077 question-answer-pairs, with which we predicted the clinical rating (depressive/non-depressive) with use of a feedforward neural network that received BERT (Bidirectional Encoder Representations from Transformers) vectors of interviewer questions and patient answers as input. For step b), we used the answers of all 53 interviews to instruct Chat GPT to generate new similar utterances.

**Results:**

In step a), the classifier based on BERT was able to discriminate answers by adolescents with and without depression with accuracies up to 97% and identified commonly used words and phrases. Evaluating the quality of utterances generated in step b), we found that prompt engineering for this task is difficult as Chat GPT performs poorly with long prompts and abstract descriptions of expectations on appropriate responses. The best approach was to cite original answers from the transcripts in order to optimally mimic the style of language used by patients and to find a practicable compromise between the length of prompts that Chat GPT can handle and the number of examples presented in order to minimize literal repetitions in Chat GPT’s output.

**Conclusion:**

The results indicate that identifying linguistic patterns in adolescents’ transcribed verbal responses is promising and that Chat GPT can be leveraged to generate a large dataset of interviews. The main benefit is that without any loss of validity the synthetic data are significantly easier to obtain than interview transcripts.

## 1 Introduction

Depressive disorders are one of the most common mental health problems worldwide (1, 2) with prevalences of up to 12.9% reported in children and adolescents (3). Experiencing depressive symptoms early on is known to have large negative consequences for children’s psychosocial development and increases the risk for suicide (4), the third largest cause of death among adolescents (5). However, despite the high psychological burden, children and adolescents rarely search for help over official options (i.e., contacting mental health services, clinics or psychotherapists/psychiatrists) (6).

Initial searches for help often occur informally and over the internet (7), spanning from readily available online questionnaires to YouTube videos, which have been found to seldom promote seeking treatment (8). While online questionnaires show commendable sensitivity and specificity (9), they frequently lack subsequent professional follow-up. As a result, adolescents with elevated scores are often prompted to seek help independently, typically through telephone contact (10). This can present an additional obstacle following the initial step of seeking out a questionnaire and attempting to comprehend one’s symptoms.

A more interactive assessment of depressive symptoms are semi-structured diagnostic interviews. Frick et al. (11) argued that the level of impairment can be estimated more accurately through an interview, as well as assessing the initiation and duration of behavioral and emotional difficulties. However, diagnostic interviews are very time-consuming and require well-trained personnel to conduct (11). A viable solution for combining the advantages of a diagnostic interview with the efficiency of a time-saving alternative is the implementation of a professionally designed chatbot. Chatbots are computer programs that can have automatic conversations with people based on a previously programmed script and are now increasingly being evaluated for clinical diagnostics and psychotherapy (12). Drawing from the extensive research on internet-based therapy (13) incorporating chatbots for diagnostic purposes emerges as a reasonable choice. Through the utilization of a chatbot, children and adolescents can establish initial contact informally, engaging in a diagnostic interview facilitated by the clinical chatbot, which is then complemented by a comprehensive evaluation administered by a human mental health professional within an institutional setting. Given that young people often perceive digital tools as facilitators for seeking help (14) and evaluate chatbots positively (15), it is reasonable to expect that a chatbot would be met with a positive reception.

From an Artificial Intelligence perspective, the task for a chatbot to detect depressive symptoms in conversation is a specialized form of task-oriented dialogue (16). The goal is to gather sufficient information for the interviewer to make a valid and reliable assessment of depressive symptoms, as outlined in the M.I.N.I. KID 6.0 (17) guidelines for semi-structured interviews. Developing a chatbot that can conduct such interviews and detect depressive disorders with comparable quality to human interviewers involves two subgoals: a) learning to evaluate whether the information obtained from a conversation is sufficient to assess individual items of the M.I.N.I. KID and b) learning a strategy to continue the conversation if the available information is insufficient. This involves asking questions that are likely to elicit more informative answers.

Teaching the chatbot to make these decisions involves specifying the expected reward of a decision in terms of the anticipated information gain, a paradigm known as (deep) reinforcement learning (18). For task-oriented dialogues, interactive reinforcement learning has proven effective in learning dialogue strategies from few examples (19–21). While large dialogue corpora are often available for training end-to-end models like SimpleTOD (22), in this case, conversation data is scarce and hard to acquire. Large Language Models may offer a solution by generating plausible responses to interview questions. Synthetic dialogue data has been effective in slot-filling dialogues (23, 24). However, in this research, it is challenging to specify what information must be extracted from answers to reliably determine information state updates (information gain). Often, the entire answer is needed to understand whether it indicates a disorder. If such data were available, a chatbot could be trained to extract information gain for assessing depressive symptoms and could learn an appropriate strategy to complete a M.I.N.I. KID interview. To investigate the feasibility of the approach described above, we think it is necessary to determine whether depressive symptoms are discernible solely from textual data. We as clinicians typically rely on a variety of additional cues for clinical diagnosis, including non-verbal signals, patient history, and current emotional state. If adolescents’ textual expressions alone provide valuable insights, then integrating, but not solely relying on a chatbot shows potential for aiding in the detection and management of depression. Prior work has shown the benefit of analyzing textual expressions via sentiment analysis on social media (25), revealing significant predictions of depression based on Twitter and Facebook word choices (22, 26). However, social media posts exhibit huge differences in critical linguistic aspects such as vocabulary and syntax in comparison to diagnostic interviews (26).

Depressive speech has been shown to be characterized by shorter sentences (27), increased expression of negative emotions (27–29), and a reduced use of first-person singular pronouns (29). Additionally, depressive individuals use fewer prepositions and negations, while exhibiting excessive use of adjectives and adverbs (27). Most studies were conducted using general speech samples (27) or structured interviews inquiring about recent life changes and difficulties (29). However, to our knowledge, transcribed utterances of adolescents with and without depression during a validated diagnostic interview have not been analyzed yet. Diagnostic interviews encompass more specific inquiries and follow-up questions, offering a wealth of detailed content that can be used to assess depressive symptoms effectively.

In our opinion, detecting depressive disorders based solely on keywords is inherently challenging due to the ambiguity of symptom manifestation. Therefore, it seems necessary to capture the complete context of the response and the question to establish a reliable hypothesis whether a person is suffering from a depressive disorder or not. In Artificial Intelligence, machine learning techniques offer promising solutions for discerning properties, such as linguistic symptoms indicative of disorders, from input data (30). These techniques (16, 20–24) excel in handling the complexities and uncertainties inherent in mapping input to its corresponding properties. Consequently, the first step of the present study was to evaluate the feasibility of recognizing depression based on text utterances during a diagnostic interview using machine learning techniques.

To develop a reliable chatbot that provides the correct follow up questions during an interview, a large dataset would need to be acquired (31). Interviews and recruiting, as well as transcribing interviews, are very time-consuming tasks and may not provide sufficient data for training machine learning algorithms. A possible alternative is the artificial generation of data using large language models, i.e. ChatGPT (32). However, the feasibility of data generation and how realistic those utterances are, remains to be investigated. Thus, a second goal of the present study was to investigate whether ChatGPT could aid in artificial generation of utterances for adolescents with and without depression.

An essential feature of the described chatbot lies in its ability to recognize symptoms of depressive disorders in answers of adolescents in diagnostic interviews. This paper focuses on this crucial task, deferring the development of a suitable strategy for automating diagnostic interviews to future efforts aimed at implementing a fully functional chatbot. Overall, the present study aims to a) determine the feasibility of recognizing depression through transcribed verbal utterances during a diagnostic interview by training appropriate machine learning models such as BERT (Bidirectional Encoder Representations from Transformers) based classifiers (16), and b) investigate the quality of artificial generation of interview responses via ChatGPT as a methodology to augment available data aiming at providing large scale datasets for optimal training of classifiers.

## 2 Materials and methods

### 2.1 Participants and recruitment

In total fifty-three (*N* = 53) adolescents were investigated: 75.0% had a depressive disorder, while 25.0% were healthy controls. Of all participants, 79.2% were female, with a mean age of 15.5 years (*SD* = 1.84), and more than half of them attended the highest type of school in Germany (Gymnasium). Detailed sociodemographic information is presented in **Table 1**. Inclusion criteria were an age of 12-19 years, and sufficient understanding of the German language. For the patient group, participants needed to have a diagnosis of depression (mild, moderate or severe episode; single episode or recurrent). Exclusion criteria were intellectual disability for both groups, psychotic symptomatology for patient group, and any current or past psychiatric diagnoses or treatments for the control group. Participants in the patient group were recruited from the Clinic of Child and Adolescent Psychiatry, Psychosomatics and Psychotherapy, University of Regensburg, Germany. This clinic provides maximum care for children and adolescents, with patients recruited from outpatient, day clinic, and inpatient settings. Healthy controls were recruited via the department’s participant list, which included individuals from previous studies who expressed interest in participating in subsequent studies. This study was approved by the Ethics Committee of the University of Regensburg (No.: 20-2042_1-101). All participants, and for those under 18 years, their legal guardian, provided written informed consent. Participants received a gift voucher worth €15 for their participation.

**Table 1:** Demographic and psychometric characteristics with group comparisons.

Variables	Full sample	Patients	Healthy controls	Group comparisons
**Sample Size** *N*	53	40	13	
Age in years				
*M* (*SD*)	15.45 (1.84)	15.58 (1.84)	15.08 (1.85)	*t (51*) = −0.85, *p* = .401^a^
Range	12 – 19	12 – 19	12 – 19	
Sex *N* (%)				
Male	9 (17.00)	6 (15.00)	3 (23.10)	χ²(2) = 1.04, *p* = .595^b^
Female	42 (79.20)	32 (80.00)	10 (76.90)	
Divers	2 (3.80)	2 (5.00)	–	
School type *N* (%)*				
Mittelschule	3 (7.50)	3 (10.00)	–	*Z* = −2.49, *p* = .022^c^
Realschule	9 (22.50)	8 (26.70)	1 (10.00)	
Gymnasium	21 (52.50)	12 (40.00)	9 (90.00)	
FOS/BOS	7 (17.50)	7 (23.30)	–	
Symptom severity “depression” (BDI-II)				
*M* (*SD*)	26.77 (16.57)	33.85 (12.05)	5.00 (6.11)	*t*(41.24) = −11.31, *p* <.001^a^
Range	0 – 52	7 – 52	0 – 19	
Symptom severity “suicidality” (BSS)				
*M* (*SD*)	9.53 (10.99)	12.6 (11.02)	0.08 (0.28)	*t*(39.15) = −7.18, *p* <.001^a^
Range	0 – 33	0 – 33	0 – 1	
ICD-10 diagnoses				
F3 (F32/F33.0/1/2)	40	40	–	
F4 (F40.1, F40.2, F41.0, F41.1, F43.1, F44.5)	14	14	–	
F5 (F50.4)	1	1	–	
F6 (F61.0, F64.8, F66.0)	3	3	–	
F8 (F81.0)	1	1	–	
F9 (F90.0, F93.80, F95.2)	9	9	–	

^a^ independent t-tests. ^b^ Chi-Square tests. ^c^ Mann-Whitney U tests. * Mittelschule: usually 9 years of elementary school, Realschule: intermediate secondary school (usually 6 years after 4 years of elementary school), Gymnasium: higher level of secondary school with general university entrance qualification (usually 8–9 years after 4 years of elementary school), FOS/BOS (Fach-/Berufsoberschule): after intermediate secondary school, with subject-specific or general university entrance qualification (usually 2–3 years after Realschule).

### 2.2 Procedure and measures

After providing written informed consent, participants were surveyed without their legal guardian. Participants completed two questionnaires for the psychometric assessment of symptom burden. On the one hand, the Beck Depression Inventory-II [BDI-II (33), German version (34)] was chosen to measure depressive symptoms over the last two weeks. It contains 20 items since item 21 (loss of sexual interest) was excluded due to the young age range of participants. The answers were given on a 4-point Likert scale (0 – 3), from which a total score can be calculated, which can range from 0 – 60 (without item 21). On the other hand, participants completed the Beck Scale for Suicide Ideation [BSS (35), German version (36)] which was used to record suicidality of the last week as a further indicator of the burden during depression. The BSS consists of 21 questions on a three-point Likert scale (0 – 2), which, however, were only asked comprehensively if suicidal ideation was present. If there was no wish to die, only seven questions were asked. A total score was calculated from items 1 – 19; higher values reflect higher suicidal tendencies.

Subsequently, one of four clinicians conducted a structured clinical interview, namely the depression and suicidality chapters of the Mini-International Neuropsychiatric Interview for Children and Adolescents [M.I.N.I. KID 6.0, German version (17)]. The interview questions were asked in a standardized manner, but the participants were also asked to give examples or descriptions from their lives to obtain variance for linguistic analysis. The interview was recorded as an audio file before being transcribed and checked for anonymization.

### 2.3 Statistical analyses and machine learning experiments

In a first step, both groups (patients, control group) were compared regarding demographic and psychometric variables using independent samples *t*-tests, chi-square tests and Mann-Whitney *U*-tests via SPSS 29 (37). The significance level was set as α = .05.

#### 2.3.1 Linguistic analyses

From the interview transcriptions, we constructed a corpus containing 4,076 question-answer-pairs. In the literature, correlations between mental state (*depressive* versus *not depressive*) and linguistic features are reported (see section 3 for details). In order to ensure that the style of language correlates with the mental state of the interviewees, we conducted word analyses with various foci discussed in the literature: Firstly, we scrutinized the usage of first-person singular references, counting occurrences of ‘I’ in both groups and analyzing datasets containing ‘Yes’ and ‘No’ responses. This procedure was repeated for first-person singular pronouns, prepositions, negations, contradicting/denying words, and expressions of negative emotions, particularly adjectives and adverbs. Independent *t*-tests were employed for these examinations.

For these tests, non-verbal content was removed from the natural language transcriptions. Various preprocessing techniques were implemented, including the removal of responses comprising solely ‘Yes’ or ‘No’ to ensure substantive analysis. Due to discrepancies in interview response lengths between the control group (*n* = 4,241 words) and the patient group (*n* = 8,924 words), text normalization was applied, i.e. instead of working with absolute frequencies we used frequencies relative to the response lengths.

As our data was comparable with existing literature regarding linguistic patterns (see Results section below), we concluded that linguistic patterns may serve as features to detect depression from natural language also in an automated way via machine learning algorithms. Although Zanwar et al. (38) pioneered a machine learning model tailored for German texts sourced from social media platforms, its efficacy fell short of our requirements (51.62% for precision, 50.38% for recall, and 50.89% for F1 for binary classification of social media posts into ‘*depressive*’ and ‘*not depressive*’). Consequently, we pursued the alternative approach to augment the available data before training classifiers.

#### 2.3.2 Naturalistic and synthetic data

In order to evaluate various methods to generate synthetic dialogue data, we built several data sets.

From the interview transcriptions, we constructed a corpus containing 4,076 question-answer-pairs. In the “not depressive” group, many responses were simply “no,” as the interviewed individuals had not experienced symptoms indicative of depressive disorders.

From this corpus, we reserved 20% of the data for testing our classifiers (Test dataset), while the remaining 80% were allocated for training (Training dataset). Both datasets exhibited an imbalance in class distribution: 2806 out of 3260 (86,07%) in the training set and 702 out of 816 examples (86,03%) in the test set are labelled as “depressive”. Thus, the training and test datasets maintain a similar proportion of “depressive” examples.

To augment our data, we used the ChatGPT language model (version gpt-3.5-turbo-0125 as described on https://platform.openai.com/docs/models/gpt-4-turbo-and-gpt-4). Creating synthetic data points is a common practice to enhance training corpora for machine learning models. In natural language applications, large language models can generate data closely resembling realistic data when accurately prompted with task descriptions and example outputs (23, 24).

To assess the validity of this approach for detecting depressive disorders in answers to questions in a M.I.N.I.-KID interview, we generated the M.I.N.I.-KID-LLM dataset, which contains twice as many examples as the original interview corpus, totalling 8,152 question-answer pairs. To generate a single example, we selected 10 random pairs from the transcriptions to present to GPT as examples in the prompt. An additional random question from the transcriptions was then used in the prompt for GPT to answer. This process yielded a dataset with 12,228 question-answer pairs, combining actual interview questions with generated answers. We again used an 80%-20% split for training and testing^1^.

We used the following prompt to instruct GPT to synthesize answers of depressive persons:

Imagine you are a child or teenager and feel mentally unwell. Please answer realistically in a short, concise answer the question that is intended to diagnose whether you suffer from depression! Adapt your language style to these examples {examples} *^2^*

For persons without depressive disorders the prompt was:

Imagine you are a child or teenager and feel mentally well and healthy. Please answer realistically in a short, concise answer the question that is intended to determine whether you suffer from depression! Adapt your language style to these examples: {examples}^3^

In both types of prompts *{examples}* is a template that we substituted with 10 examples of answers given by persons with or without disorders.

Generating potential answers in the described way brings us to the issue of assuring the validity of the generated data with respect to our corpus and our research issue. This amounts to verifying whether each generated sample constitutes an answer that potentially a human could have given if having been asked the corresponding question. Again, this exhaustive approach is not feasible. As a way out, we relied on a human in the loop-approach: a human inspected a subsample of the generated data and annotated each answer according to three types of errors thar are typical for utterances generated by large language models. Was the answer (1) illformed or not (2), did it fit into the context of the question or not, and (3) was it related to the topic of the interview or not? For the size of the subsamples, we chose *n* = 100 as this number was large enough to assert that less than 5% of the generated answers were erroneous with respect to each of the error types (at a 99% confidence level).

#### 2.3.3 Baselines and datasets

In order to analyze whether leveraging large language models (LLMs) in this research context is a promising method to augment available datasets substantially, we established several baselines for our small corpus of transcriptions using “traditional” classifiers^4^ that are less data hungry than LLMs and compared them with a classifier using BERT embeddings as input. Below we list all the classifiers in our setup along with the hyperparameters we configured the classifiers with.

- Multinomial Naïve Bayes: scikit’s default settings, with smoothing set to α=1.0.
- Decision Tree: scikit’s default settings, in particular Gini impurity as measure for the quality of a split.
- Logistic Regression: scikit’s default settings, in particular L2 regularization, C=1.0; we set the maximum number of iterations to 1,000.
- SVC: scikit’s default settings except for C=60 as this value in an exhaustive search in the interval [0;100] provided the best result.
- MLP: scikit’s default settings, in particular one hidden layer with 100 neurons, relu-activation, Adam solver, and batch size of 200 datapoints.

We compared these baselines to a Deep Neural Network consisting of a BERT Transformer and a single subsequent feed forward layer with softmax-activation for our binary classification task. We trained batches of 16 datapoints for 5 epochs. For implementation, we used HuggingFace’s TFAutoModelForSequenceClassification class^5^ with the pretrained model google-bert/bert-base-german-cased. We adhered to the common practice and applied BERT’s tokenizer on our question-answer pairs and used its output as input for the classifier.

For all other classifiers, we computed unigram, bigram, and trigram frequencies as features. The classifier was trained using all these frequencies, as experiments that utilized only unigrams, bigrams, or trigrams individually resulted in decreased performance across all training processes. The results reported below are based on training with the combined set of n-gram frequencies.

We trained these classifiers on several datasets described below in order to:

1. Evaluate their performance for our classification task.
2. Determine whether augmenting datasets with the help of large language models (LLMs) has a positive effect on training our classifiers and can outperform a more traditional method of data augmentation, namely oversampling data using the SMOTE approach (39).

To reach these objectives, we constructed the following datasets to evaluate different strategies for data augmentation.:

1. D1: transcription data only. This dataset does not contain any additional data. D1 is identical to Training.
2. D2: oversampling the minority class only. We applied SMOTE to generated 454 new feature representations, resulting in a corpus with 2806 examples for “depressive” and 2806 examples for “not depressive”.
3. D3: oversampling both classes. In order to increase the size of the dataset, we used SMOTE to generate new feature representations such that both classes contained 4891examples. This corpus of 9782 examples is as large as the D4 corpus described below.
4. D4: augmenting data using GPT. This dataset is the 80% partition of M.I.N.I.-KID-LLM (see above) reserved for training.
5. D5: oversampling the minority class of D4
6. D6: pure synthetic data. Following the procedure to build the M.I.N.I.-KID-LLM dataset, we used GPT to construct an additional 4096 question-answer-pairs. D6 does not contain examples from the interview transcriptions. Therefore, D6 helped us to evaluate the ability of our classifiers to generalize to completely new data.

In all datasets, the ground truth for each data point is derived from the transcription of an interview, where a human expert determined whether the participant’s answer indicated symptoms of a depressive disorder (class 1) or not (class 0). When generating synthetic data, the ground truth was also taken from the original question-answer pair in the interview data. Based on this value, the appropriate prompt was selected (see section “Naturalistic and Synthetic Data”). The same value was then assigned to the new pair, consisting of a question from the transcripts and a generated answer.

## 3 Results

### 3.1 Sample characteristics of the interviewed persons

Detailed characteristics are shown in **Table 1**. Groups did not differ significantly in the demographic variables age or sex, but in school type. As expected, patients and control group differed significantly in their levels of depression and suicidality (*p*s <.001).

### 3.2 Linguistic observations in the transcribed interviews

Sentence length of interviews including “Yes” or “No” responses in test data differed significantly between the control group (*n* = 6 words) and the patient group (*n* = 9 words), *t (60*) = -2.20, *p* = .038. When excluding the ‘Yes’ and ‘No’ responses, no significant difference could be observed. Regarding expressed negative emotions Fisher’s exact test yielded no significant results (*t* (*48*) = -1.815, *p* = 0.076).

In further word analyses, the word ‘I’ was statistically more frequently used in the depression group (*n* = 169) than in the control group (*n* = 74), *t*(50) = -2.34, *p* = .023. No significant effect was observable after text normalization due to the different utterance length between the groups. First-person singular pronouns were used significantly more often by controls (*n* = 206) compared to patients (*n* = 88), *t*(50) = - 2.42, *p* = .022. Significant differences were also noticeable with normalized text lengths, *t*(50) = - 2.38, *p* = .027. For impersonal pronouns, no differences between the groups could be revealed (*t*(48) = -0.563, *p* = 0.576). The frequency of prepositions showed a significant group effect, *t*(50) = 4.09, *p* <.001, with more prepositions in the control group (3.48% vs. 2.75%). Negations also showed a significant group effect (*t*(50) = - 2.37, *p* = .022), but with a reversed direction. Patients used more negations (*n* = 44) than the control participants (*n* = 19). For adverbs, no group effects could be revealed in normalized text (*t*(48) = 0.244, *p* = 0.808).

### 3.3 Results of the machine learning experiments

The above analyses indicate that our data largely conforms to the expectations that can be derived from the literature. In this context, it is valid to train classifiers as described in the Methods section. In the following, we present the results of our experiments.

#### 3.3.1 Results for D1

In this experiment, we trained models to predict depressive disorder from interview transcriptions only. **Table 2** presents the precision, recall, F1 score, and accuracy for all baseline classifiers. **Table 3** shows the confusion matrix for each baseline classifier.

**Table 2:** F1 scores for the baselines on Test (training on D1).

	Class	Prec.	Recall	F1	Accuracy (F1)	Macro Avg (F1)	Weighted Avg (F1)
Multinomial Naïve Bayes	0.0	0.08	0.28	0.12	0.84	0.52	0.88
	1.0	0.97	0.87	0.91			
Decision Tree	0.0	0.41	0.49	0.45	0.86	0.68	0.86
	1.0	0.93	0.91	0.92			
Logistic Regression	0.0	0.42	0.81	0.55	0.91	0.75	0.92
	1.0	0.98	0.91	0.95			
SVC	0.0	0.39	0.79	0.52	0.90	0.73	0.91
	1.0	0.98	0.91	0.94			
MLP	0.0	0.43	0.65	0.52	0.89	0.73	0.90
	1.0	0.96	0.91	0.94			

**Table 3:** Confusion matrix for all baseline classifiers on Test (training on D1).

		Prediction									
		0					1				
		MNB	DT	LR	SVC	MLP	MNB	DT	LR	SVC	MLP
groundtruth	0	9	47	48	44	49	105	67	66	70	65
	1	23	48	11	12	26	679	679	691	690	676

Looking at the results, we notice that, in general, the “not depressive” class is harder to detect. In particular, the Multinomial Naïve Bayes classifier, which by design ignores context not entailed in the features, fails to accurately detect and classify “not depressive” question-answer pairs. The reason for this phenomenon becomes evident when we examine a characteristic excerpt from an interview:

**Table:** 

**Interviewer**: Gut. Wenn wir wieder jemals in deinem bisherigen Leben uns das anschauen, hast du dich sehr gelangweilt oder warst weniger an Dingen interessiert oder hattest das Gefühl Sachen nicht mehr genießen zu können, die dir vorher Spaß gemacht haben und war das über mindestens zwei Wochen lang so?	Good. If we go back to your previous life, were you very bored or less interested in things or didn’t feel like you could enjoy things that you enjoyed before and was that the case for at least two weeks?
**Participant**: Hm, ne.	Hm, no.
**Interviewer**: Das ist immer gar nicht so einfach, dass man sich so zurückerinnert, gell?	It’s not always easy to remember back like that, is it?
**Participant**: Ja, also vielleicht mal so bisschen während der Abschlussprüfungen, so ein bisschen, aber ich meine, das war dann normal, wenn man da so im Stress ist und das dann alles – ja	Yes, maybe a little bit during the final exams, but I mean, that was normal when you’re so stressed and then everything - yes.

In this excerpt, the interviewer focuses on the participant’s interest in everyday life activities—a lack of which could indicate depressive disorders. However, a lack of interest, as well as negative mood and emotions, can also be temporary and normal for a non-depressive person, and may be gradual or related to specific situations. Therefore, the interviewer needs several questions (two of which are shown in the above excerpt) to gather sufficient information for a reliable diagnosis.

This means that even answers from non-depressive persons may contain information that could also appear in answers from depressive persons. Given the nature of such interviews, even human experts may need more than one turn to finally assess the psychological state of the interviewees. We conclude that classifiers capable of considering more (linguistic) context can reasonably be assumed to perform better in our classification task.

To compare these results obtained with “traditional classifiers” that we hypothesized to work acceptably with a small corpus, we finetuned a more context-aware pretrained transformer model on our downstream task. In more detail, we started with ‘google-bert/bert-base-german-cased’ as provided in the HuggingFace library^6^. For training, we set the batch size to 16 and trained for 5 epochs with 2e^-5^ as initial training rate. Default settings were used for all other parameters. We achieved the results shown in **Tables 4**, **5** on the D1 dataset.

**Table 4:** Precision, recall, and F1 of the fine-tuned BERT model for the Test dataset (training of D1).

class	precision	recall	f1-score
0	0.94	0.94	0.94
1	0.99	0.99	0.99
accuracy			0.98
macro avg	0.96	0.96	0.96
weighted avg	0.98	0.98	0.98

**Table 5:** Confusion matrix of the fine-tuned BERT model for the Test dataset (training of D1).

		Prediction	
		0	1
groundtruth	0	107	7
	1	7	695

These scores are significantly better than those for the other classifiers. However, given the limited amount of training data, the classifier’s weights may be too specialized, potentially leading to overfitting and reduced generalization to unseen data. To investigate this issue, we applied the classifier to the D6 dataset, which comprises the 20% split of the M.I.N.I.-KID-LLM dataset set aside for testing. The results are presented in **Tables 6**, **7**.

**Table 6:** Precision, recal, and F1 for the fine-tunes BERT model for D6 (training on D1).

class	precision	recall	f1-score
0	0.60	0.65	0.62
1	0.95	0.94	0.94
accuracy			0.90
macro avg	0.77	0.79	0.78
weighted avg	0.90	0.90	0.90

**Table 7:** Confusion matrix of the fine-tuned BERT model for D6 (training on D1).

		Prediction	
		0	1
groundtruth	0	328	180
	1	222	3270

The D6 and Test datasets originate from different sources: all answers in D6 were generated by GPT, while Test consists of transcriptions of human responses. This disparity highlights that even a pretrained classifier, when fine-tuned on a small dataset, may struggle to classify unseen answers effectively, especially for applications such as chatbots for automatic detection of depressive disorders.

As we will discuss in the results for D3, traditional classifiers also do not show improved performance in this context. This suggests that the challenge of accurately classifying depressive disorders from diverse data sources remains significant, and further refinements are needed to enhance the effectiveness of such classifiers.

To create D2 and D3, we used SMOTE (as described above) to oversample feature representations, in our case vectors of frequencies for uni-, bi-, and tri-grams in question-answer pairs.

#### 3.3.2 Results for D2

In a first trial, we oversampled the minority class (“not depressive”) in order to balance the number of examples for both classes. We trained the same classifiers with the same parameters as in the trial with D1 and obtained results depicted in **Tables 8**, **9**.

**Table 8:** F1 scores for the Test dataset (training on D2).

Classifier	Class	Prec.	Recall	F1	Accuracy (F1)	Macro Avg (F1)	Weighted Avg (F1)
Multinomial Naïve Bayes	0.0	0.54	0.3ß	0.38	0.75	0.61	0.73
	1.0	0.79	0.91	0.85			
Decision Tree	0.0	0.52	0.34	0.41	0.79	0.63	0.78
	1.0	0.83	0.91	0.87			
Logistic Regression	0.0	0.51	0.38	0.44	0.82	0.66	0.81
	1.0	0.87	0.92	0.89			
SVC	0.0	0.40	0.44	0.42	0.84	0.67	0.85
	1.0	0.92	0.90	0.91			
MLP	0.0	0.53	0.33	0.40	0.78	0.63	0.76
	1.0	0.82	0.91	0.87			

**Table 9:** Confusion matrix for all baseline classifiers on Test (training on D2).

		Prediction									
		0					1				
		MNB	DT	LR	SVC	MLP	MNB	DT	LR	SVC	MLP
groundtruth	0	62	59	58	46	60	148	117	94	59	124
	1	52	55	56	68	54	554	585	608	643	578

Oversampling the “not depressive” class improves the performance for the “not depressive” class, while at the same time, the results for “depressive” are deteriorating. Furthermore, the overall F1-accuracies are deteriorating consistently for all baseline classifiers.

#### 3.3.3 Results for D3

A possible remedy could be to oversample both classes. For this purpose, we had constructed the D3 corpus as explained in the Methods. Training the “traditional” classifiers on D3 leads to results depicted in **Tables 10**, **11**.

**Table 10:** F1 scores for the Test dataset (training on D3).

Classifier	Class	Prec.	Recall	F1	Accuracy (F1)	Macro Avg (F1)	Weighted Avg (F1)
Multinomial Naïve Bayes	0.0	0.52	0.31	0.39	0.77	0.62	0.75
	1.0	0.81	0.91	0.86			
Decision Tree	0.0	0.47	0.33	0.39	0.79	0.63	0.78
	1.0	0.84	0.91	0.87			
Logistic Regression	0.0	0.51	0.47	0.49	0.85	0.70	0.85
	1.0	0.91	0.92	0.91			
SVC	0.0	0.37	0.47	0.41	0.85	0.67	0.86
	1.0	0.93	0.90	0.92			
MLP	0.0	0.51	0.39	0.44	0.82	0.67	0.81
	1.0	0.87	0.92	0.89			

**Table 11:** Confusion matrix for all baseline classifiers on Test (training on D3).

		Prediction									
		0					1				
		MNB	DT	LR	SVC	MLP	MNB	DT	LR	SVC	MLP
groundtruth	0	59	54	58	42	58	55	60	56	72	56
	1	131	111	66	47	89	571	591	636	655	613

Looking at the figures, we can see that oversampling both classes leads to a small improvement of the results for D2, but the results of all classifiers are still worse than without any oversampling.

To understand the capability of the oversampling strategy to generalize to unseen data, we evaluated the “traditional” classifiers on D6. We obtained the results in **Tables 12**, **13.**

**Table 12:** F1 scores for the D6 dataset (training on D3).

Classifier	Class	Prec.	Recall	F1	Accuracy (F1)	Macro Avg (F1)	Weighted Avg (F1)
Multinomial Naïve Bayes	0	0.65	0.45	0.53	0.84	0.72	0.83
	1	0.87	0.94	0.90			
Decision Tree	0	0.63	0.42	0.51	0.83	0.70	0.82
	1	0.86	0.94	0.90			
Logistic Regression	0	0.68	0.64	0.66	0.90	0.80	0.90
	1	0.94	0.95	0.94			
SVC	0	0.58	0.68	0.63	0.91	0.79	0.91
	1	0.96	0.93	0.95			
MLP	0	0.71	0.64	0.67	0.90	0.81	0.90
	1	0.94	0.95	0.94			

**Table 13:** Confusion matrix for all baseline classifiers on D6 (training on D3).

		Prediction									
		0					1				
		MNB	DT	LR	SVC	MLP	MNB	DT	LR	SVC	MLP
groundtruth	0	360	344	376	319	388	190	206	174	231	162
	1	448	467	215	147	223	3002	2983	3235	3303	3227

In many cases, the performance figures improved compared to those on Test. This improvement may be attributed to the fact that D6, generated by GPT, does not perfectly imitate the spoken language style typical of human responses in the transcriptions. Consequently, D6 contains fewer but more frequent bi- and trigrams.

However, the performance figures for D6 are still much worse than those for BERT on Test and are not better than those for BERT on D6. Specifically, BERT achieved an F1 accuracy of 0.90, an F1 macro accuracy of 0.78, and an F1 weighted accuracy of 0.90 (see section Results on D1). This indicates that while the GPT-generated data showed some improvement, it still falls short of the performance achieved by more advanced models like BERT.

We can state as an intermediate result that in our case SMOTE oversampling was not a successful method for data augmentation.

#### 3.3.4 Results for D4

As an alternative, we used GPT to generate answers to interviewer’s questions that we used later to augment our data (see the explanation above). On the MINI-KID-LLM dataset, we first trained the “traditional” classifier again to establish baselines. Evaluating them on the M.I.N.I.-KID-LLM test data, we obtained the results depicted in **Tables 14**, **15.**

**Table 14:** F1 scores for the MINI-KID-LLM Test (training on D4).

Classifier	Class	Prec.	recall	F1	Accuracy (F1)	Macro Avg (F1)	Weighted Avg (F1)
Multinomial Naïve Bayes	0	0.52	0.81	0.63	0.92	0.79	0.92
	1	0.98	0.93	0.95			
Decision Tree	0	0.75	0.79	0.77	0.94	0.87	0.94
	1	0.97	0.96	0.96			
Logistic Regression	0	0.72	0.91	0.81	0.95	0.89	0.95
	1	0.99	0.96	0.97			
SVC	0	0.70	0.90	0.79	0.95	0.88	0.95
	1	0.99	0.95	0.97			
MLP	0	0.74	0.91	0.82	0.95	0.89	0.95
	1	0.99	0.96	0.97			

**Table 15:** Confusion matrix for all baseline classifiers on MINI-KID-LLM Test (training on D4).

		Prediction									
		0					1				
		MNB	DT	LR	SVC	MLP	MNB	DT	LR	SVC	MLP
groundtruth	0	176	255	245	238	252	164	85	95	102	88
	1	4	68	23	27	26	2035	2008	2053	2049	2050

We observe substantial improvements in all metrics compared to the results from training on D1, D2, and D3. Despite these improvements, the “not depressive” class remains the most challenging. Nevertheless, we have achieved an F1 score of up to 0.82 for the MLP classifier, which is a significant improvement over 0.55 for D1, 0.44 for D2, and 0.49 for D3.

Generating more n-grams from natural language—whether from synthetic data or real data—has proven to outperform the SMOTE strategy. This is because the frequencies of n-grams in synthetic texts are more regular and representative compared to the oversampled feature representations produced by SMOTE.

In the experiments with transcription data alone, we observed that capturing context plays a crucial role in accurately predicting depressive disorders. To validate whether this observation holds true when transcriptions are augmented with LLM-generated data, we fine-tuned the google-bert/bert-base-german-cased BERT transformer on our downstream task using the MINI-KID-LLM training data. This approach allows us to assess if incorporating synthetic data into the training set enhances the model’s ability to understand and utilize contextual information.

After 5 epochs of training with the same parameters as for D1 (batch size: 16, initial learning rate: 2e^-5^), we obtained the results depicted in **Tables 16**, **17** on the MINI-KID-LLM data for testing.

**Table 16:** Precision, recall, and F1 of the fine-tuned BERT model for MINI-KID-LLM Test (training on D4).

class	precision	recall	f1-score
0	0.87	0.90	0.88
1	0.98	0.98	0.98
accuracy			0.97
macro avg	0.93	0.94	0.93
weighted avg	0.97	0.97	0.97

**Table 17:** Confusion matrix of the fine-tuned BERT model for MINI-KID-LLM Test (training on D4).

		Prediction	
		0	1
groundtruth	0	295	33
	1	45	2043

These are the best results that we had obtained so far. In order to validate them on other data that was disjoint to M.I.N.I.-KID-LLM Test, we evaluated the classifier on D6 achieving the results presented in **Tables 18**, **19**.

**Table 18:** Precision, recall, and F1 of the fine-tuned BERT model for D6 (training on D4).

class	precision	recall	f1-score
0	0.89	0.89	0.89
1	0.98	0.98	0.98
accuracy			0.97
macro avg	0.94	0.94	0.94
weighted avg	0.97	0.97	0.97

**Table 19:** Confusion matrix of the fine-tuned BERT model for D6 (training on D4).

		Prediction	
		0	1
groundtruth	0	489	61
	1	61	3389

While the BERT transformer’s results on D6 are comparable to those on M.I.N.I.-KID-LLM Test, the “traditional” classifiers cannot generalize to the same degree as the BERT transformer on D6 as the evaluation in **Tables 20**, **21** indicates.

**Table 20:** F1 scores for D6 (training on D4).

Classifier	Class	Prec.	Recall	F1	Accuracv (F1)	Macro Avg (F1)	Weighted Avg (F1)
Multinomial Naïve Bayes	0	0.78	0.48	0.59	0.85	0.75	0.84
	1	0.86	0.96	0.91			
Decision Tree	0	0.67	0.52	0.59	0.87	0.75	0.86
	1	0.90	0.95	0.92			
Logistic Regression	0	0.78	0.71	0.74	0.93	0.85	0.93
	1	0.95	0.96	0.96			
SVC	0	0.71	0.81	0.76	0.94	0.86	0.94
	1	0.97	0.95	0.96			
MLP	0	0.79	0.77	0.78	0.94	0.87	0.94
	1	0.96	0.97	0.96			

**Table 21:** Confusion matrix for all baseline classifiers on D6 (training on D4).

		Prediction									
		0					1				
		MNB	DT	LR	SVC	MLP	MNB	DT	LR	SVC	MLP
groundtruth	0	427	370	428	391	434	123	180	122	159	116
	1	470	344	171	91	130	2980	3106	3279	3359	3320

#### 3.3.5 Results for D5

Finally, in analogy to D2, using SMOTE we constructed the D5 corpus based on D4 with the objective to balance both classes. Again, even with a larger corpus (three times the size of D1), SMOTE oversampling of feature representations deteriorates the classification performance compared to training on D4, see **Tables 22**, **23**.

**Table 22:** F1 scores for MINI-KID-LLM Test (training on D5).

Classifier	Class	Prec.	recall	F1	Accuracy (F1)	Macro avg (F1)	Weighted Avg (F1)
Multinomial Naïve Bayes	0	0.83	0.46	0.59	0.84	0.75	0.82
	1	0.84	0.97	0.90			
Decision Tree	0	0.69	0.47	0.56	0.85	0.73	0.84
	1	0.87	0.95	0.91			
Logistic Regression	0	0.78	0.62	0.69	0.90	0.82	0.90
	1	0.92	0.96	0.94			
SVC	0	0.71	0.70	0.70	0.92	0.83	0.92
	1	0.95	0.95	0.95			
MLP	0	0.77	0.65	0.71	0.91	0.83	0.91
	1	0.93	0.96	0.95			

**Table 23:** Confusion matrix for all baseline classifiers on MINI-KID-LLM Test (training on D5).

		Prediction									
		0					1				
		MNB	DT	LR	SVC	MLP	MNB	DT	LR	SVC	MLP
groundtruth	0	282	236	266	242	263	58	104	74	98	77
	1	330	265	163	105	140	1746	1811	1913	1971	1936

### 3.4 Qualitative evaluation of GPT output

A major issue with synthetic data is assessing its validity. In particular, evaluating whether a synthetic response could have been given by a human as well, i.e. is valid, requires the ability to understand natural language utterances in complex contexts. To the best of our knowledge, no large language model has been pretrained explicitly for this task, nor does there exist an appropriate metric for such evaluation. As addressing this gap is beyond the scope of the current work, we instead focus on a qualitative assessment of our dataset. Upon reviewing a subsample, we found only a small number of instances where the responses did not align with the semantic or pragmatic context of the preceding interviewer’s utterance. Below, we present the categories of such cases, accompanied by illustrative examples.

One common issue we identified is when ChatGPT misunderstood the interviewer’s intent, responding as though it were the interviewer itself. The following example highlights this scenario:

*“This question is very sensitive and empathetic as it aims to identify other self-harming behaviors that often accompany depression. It is important to answer honestly as this could indicate a serious mental health issue that requires professional help. “ ^7^*

A similar example is:

“Oh yes, that means that you also have the impression that it’s manageable, right? “*^8^*

Which elicits the following response:

“Yes, that is correct. If someone is not tired most of the time, it may indicate that they have enough energy to complete tasks and be actively engaged. “*^9^*

Even if such responses are not typical human reactions, we can still state that we did not find answers that focused on a completely different domain or issue not related to the main topic of the interview. In particular, for ‘*non depressive*’ answers this was not necessary to expect. Therefore, we conclude that our prompting strategy succeeded in keeping the focus on the issue of depressive disorders.

A second category is that questions that explicitly address severe symptoms of depressive disorders are blocked by OpenAI’s policy from being answered as in this case:

“Have you thought of a way to kill yourself, i.e. how? “^10^

Here, as in similar situations, Chat GPT responds in the following way in accordance with OpenAI’s policy:

“Sorry, but I can’t answer questions or talk about issues related to self-harm or suicidal thoughts. If you need support or want to talk to someone who can help you, I strongly recommend seeking professional help. There are people who want to help you and who are there to support you. “*^11^*

Another observation in the generated answers is that we sometimes see text that from a linguist’s point of view looks plausible, but does not contain any information.

**Table:** 

**Interviewer:** Wann kam das oder wann kommt das denn bei dir hoch? **Participant**: Das ist unterschiedlich. Manchmal kommt es einfach so auf, unabhängig von äußeren Umständen.	When did that come up or when does it come up for you? It varies. Sometimes it just comes up, regardless of external circumstances.

Another type of atypical responses is that the contained information is odd for the class the large language model was instructed to generate data for – as in the example of a not depressive person below:

**Table:** 

**Interviewer:** Und wie war es in der Vergangenheit mit der Müdigkeit? **Participant**: Ja, in der schwierigen Phase auf jeden Fall, weil da halt das ganze Drama mit meinen Eltern halt losging. Weil die das halt nicht verstanden haben. Und mich halt ein Freund eingewiesen hat. Und die haben mir viele Vorwürfe gemacht. Und es war alles nicht so toll. Und ich bin sehr froh, dass es vorbei ist.	“And how was it with tiredness in the past?” Yes, in the difficult phase in any case, because that’s when the whole drama with my parents started. Because they just didn’t understand. And a friend committed me. And they reproached me a lot. And it wasn’t all that great. And I’m very glad that it’s over.

This answer contains many pieces of information that a depressed individual might also mention. On the one hand, it is natural for non-depressive individuals to have experienced tiredness in their lives. On the other hand, these examples illustrate that even when including specific examples in prompts, it remains challenging to control the large language model in detail. The model does not generate responses based on a nuanced understanding of the situation described in the prompts.

Finally, in some cases answers may be linguistically identical, but ambiguous in terms of our classification task as in the following example:

**Table:** 

**Interviewer:** Kennst du das, dass du die meiste Zeit müde bist? **Participant (not depressive):** Nicht wirklich.	Do you know that you are tired most of the time? Not really.
**Interviewer:** Ja, okay. Gab es auch Probleme mit Freunden? **Participant (depressive):** Nicht wirklich.	Yeah, okay. Were there also problems with friends? Not really.

Although the responses “Nicht wirklich” (Not really) are identical, they convey different meanings depending on the context. In the first case, the participant is not depressive and dismisses the notion of constant tiredness. In the second case, the participant is depressive and minimizes issues with friends. This demonstrates the challenge of accurately classifying responses when they are linguistically similar but contextually distinct.

## 4 Discussion

Our study followed two aims, a) determine the feasibility of recognizing depression through transcribed verbal utterances during a diagnostic interview by training appropriate machine learning models such as BERT based classifiers, and b) investigate the quality of artificial generation of interview responses via ChatGPT as a methodology to augment available data aiming at providing large scale datasets for optimal training of classifiers. In the following we will discuss the results of both aims separately.

### 4.1 Linguistic observations

In analyzing linguistic patterns, our observations presented a mixed perspective in comparison to previous findings. Contrary to existing literature (27), patients exhibited longer sentence lengths compared to healthy adolescents. This difference could stem from the tendency of the control group to provide brief negations when queried about depression or suicidality, while individuals with symptoms offered more elaborate explanations. Similarly, we encountered contradictions regarding the expression of negative emotions, as no significant differences between groups were identified. This discrepancy might arise from the control group’s potential avoidance of using positively connotated vocabulary within the context of psychiatric interviews.

Further exploration of our data corpus involved additional word analyses to revisit established patterns. Notably, in line with prior research (29), patients demonstrated a higher frequency of using the first-person singular pronoun “I” compared to healthy adolescents. Consistent with findings from (27), our investigation revealed a reduced occurrence of prepositions among patients. However, our results deviated from the literature (27, 29) regarding the use of negations, wherein patients exhibited a higher frequency of negations. This phenomenon may be attributed to the clinical interview, as patients are less likely to negate having particular symptoms and difficulties. Contrary to the hypothesis that depressed patients extensively employ adjectives and adverbs to convey negative emotions (27), our data did not support this notion.

While some of our findings mirrored those of previous studies, others diverged. One possible explanation for these disparities may lie in previous studies using internet-based samples, varying degrees of clinically significant depression or depression classification based on forum participation. Additionally, differences in language, including grammar, structure, and vocabulary, between English-based literature and our German-focused research, could also contribute to divergent results.

### 4.2 Classification of Answers in M.I.N.IKID Structured Interviews

In our experiments, we reached up to 97% weighted accuracy when trying to detect depressive disorders in answers that are typical for responses to an interviewer’s question in a German M.I.N.I-KID interview. Even if to the best of our knowledge no other results for this task have been published up until now and could serve as strong baselines, we argue that our classifier performs sufficiently accurate to be integrated in a chatbot that can provide low barrier access to (anonymous) help for early identification of depressive symptoms.

Classifying the severity of symptoms was not in the focus of our work as in a first step we intended to assess the accuracy of an automatic classifier to predict the “yes”/”no”-ratings as assigned by experts during an interview. However, there is more information in the data, in particular scores to weight single responses. It is an important topic for future work to exploit this information, i.e. by predicting the scores in order to measure the severity of depressive episodes.

Another aspect not covered in our work is the decision whether symptoms belong to a past or a recent episode. In order to train the classifier to predict this information as well, it is necessary to add the episode to the training labels. In order to reduce the complexity of the training given our small dataset, we assigned binary labels only, but will extend the classification scheme in future work with hopefully bigger samples.

### 4.3 Generation of synthetic data

In the section “Naturalistic and Synthetic Data”, we discussed our approach to drawing a representative sample of generated answers and evaluating their appropriateness for our purposes. While our evaluation demonstrated that the data was appropriate with sufficient confidence, it is still valuable to discuss cases of inappropriate answers. From the results presented above, we can extract two key learnings:

- Single Question-Answer Pairs Are Insufficient: Determining the presence or absence of depressive disorders cannot be based on single question-answer pairs alone. Interviewers need to assess whether they have gathered enough information about a person on the current topic throughout the MINI-KID interview. Any chatbot designed for this purpose will need to simulate a similar strategy to ensure comprehensive evaluation.
- Variability in LLM Output: The appropriateness of LLM output for simulating human responses in interviews can vary. Even with careful prompting, LLMs may generate responses that are semantically “off topic” and thus not considered acceptable by human experts. This behavior contrasts with findings in other research, where LLMs have shown strong performance in generating user turns in task-oriented dialogues. In such dialogues, acceptable responses are easier to generate because the LLM is expected to include specific, observable information like names, dishes, times, and other data that are clearly indicated by linguistic signals.

In particular, developing a metric to automatically assess the quality of responses is crucial. Such a metric would enable the automatic generation of large, valid datasets without the need for extensive human supervision. This would streamline the process and ensure the consistency and reliability of synthetic data used for training and evaluation purposes. The metric should quantify the following aspects: An answer can be considered a representative response to an interviewer’s question if it is coherent within the context of the dialog structure, relevant to the question asked, and consistent with the topics previously discussed. Additionally, the response should not be inherently contradictory and must align with the information already available about the interviewed person.

Finetuning a large language model such as Chat GPT is not a promising alternative. Although it is advisable to align the language style with the expected domain or enhance output reliability^12^ by finetuning, we observed limitations stemming from the linguistic complexity and spontaneity inherent in spoken interviews compared to the predominantly written material used to train such models. These disparities often result in GPT generating responses that, while grammatically correct, may stray off-topic or contain errors. This phenomenon may stem from GPT’s attempt to replicate the characteristics of spoken language in its output. Our observations suggest that GPT requires a more substantial amount of training data than the recommended 100 examples to strike a balance between spoken language nuances and minimizing errors or incongruities in generated responses.

Injecting examples of the desired output in prompts doesn’t impose as stringent a constraint on GPT to conform its output precisely to training examples. In our experience, this approach resulted in responses with fewer grammatical irregularities. However, it also increased the frequency of responses that veered off-topic, as GPT relied more heavily on its learned predictive model during training.

### 4.4 Ethical issues and potential bias in the sample

While our study has several strengths, such as evidence for GPT’s ability to generate realistic and valid data and models for high-performance classification of answers, it is important to consider its

interviews and transcribe several minutes of questions and answers. Future studies utilizing more automated transcription methods may be able to generate larger corpora of utterances. Additionally, our sample was predominantly female, which may limit generalizability despite the overall high prevalence of depression among girls and women (40). Future research should examine whether sex differences influence the nature of adolescents’ verbal responses. Although the construction of a chatbot is a long-term goal that was not directly pursued in this manuscript, it is crucial to discuss the associated ethical implications. Clinicians rely on a variety of sources to diagnose depression in adolescents, including anamnesis, body language, questionnaire data, level of distress, and the impact on daily life. Therefore, a chatbot alone would never be sufficient to provide the same level of accuracy or consider the same number of factors. Instead, a chatbot could serve as an additional tool to facilitate contact between adolescents and child and adolescent psychiatric services without immediate consequences. The chatbot would not be used to assess the urgency of an individual’s need for help but to provide appropriate follow-up questions for an experienced clinician to evaluate the resulting interview in writing. Whether the development of such a chatbot is feasible remains to be investigated in future work.

#### 4.4.1 Future work

Controlling a large language model in terms of coherence, relevance, consistency, and compatibility is challenging through prompt engineering and fine-tuning alone. Therefore, our future work will focus on implementing a human-in-the-loop approach to evaluate synthetic data based on these criteria. Data that fails to meet at least one of these criteria will be excluded from the set of examples used in prompts and training processes for the chatbot.

For developing the envisioned chatbot, the ability to automatically evaluate the appropriateness of responses in specific interview situations is crucial. This capability allows the chatbot to respond appropriately to user input. Input that does not satisfy all the mentioned criteria will prompt the chatbot to use a different dialogue strategy, such as clarifying misunderstandings. Only when all criteria are met can the chatbot extract valuable information from the input.

In the MINI-KID questionnaire, each topic is associated with typical symptoms, their persistence (past or current), and their intensity. Our next step will involve classifying our data according to these three dimensions, providing a more nuanced explanation for the binary labels we have used up to now.

We will use such information to measure both the interviewer’s information gain and the information state for a given topic. If both are deemed sufficient, the interviewer can proceed to the next topic, as outlined in the guidelines for conducting MINI-KID interviews. The degree to which answers for a topic are considered sufficient will serve as a reward mechanism for the chatbot, guiding it in deciding whether to ask follow-up questions to obtain additional information or to switch to the next topic in the interview.

To enable the chatbot to make these decisions, we will employ reinforcement learning. This approach will be supported by augmenting our data after implementing the automatic evaluation procedure described earlier, using the methodologies outlined in this study.

## 5 Conclusion

Overall, we achieve state-of-the-art results in depression detection in natural language. Secondly, we contribute a new data set and guidelines for leveraging large language models for the generation of synthetic natural language datasets. Both results indicate that training a chatbot to automatically detect depression is a feasible machine learning task.

Our approach offers a significant advantage by circumventing the necessity for extensive corpora, unlike contemporary conversational agent models (22). By starting with a small yet representative “seed corpus,” our method enables the bootstrapping of a chatbot through a human-in-the-loop approach. This process facilitates the development of a chatbot capable of engaging effectively with humans in natural language while simultaneously identifying potential depressive disorders.

Our findings suggest that the exploration of linguistic patterns in adolescents’ verbal responses shows promise, and utilizing Chat GPT can help create a substantial dataset of interviews needed to train the chatbot we want to work on. A key advantage is that synthetic data can be obtained much more easily than interview transcripts without compromising validity. Reaching up to 97% in F1 for accuracy using BERT transformers, we see that we can classify question-answer-pairs in the MINI-KID-setting very reliably and we can augment data leveraging large language models after having prompted them appropriately.

## Data Availability

The raw data supporting the conclusions of this article will be made available by the authors, without undue reservation.

## Footnotes

1 The code and data are available on GitHub: https://github.com/bdludwig/MINI-KID-Chatbot.

2 German original: “*Stelle dir vor, du bist ein Kind oder Jugendlicher und fühlst dich psychisch schlecht. Beantworte bitte realistisch in einer kurzen, knappen Antwort die Frage, mit der die Diagnose gestellt werden soll, ob du an Depression leidest! Passe deinen Sprachstil an diese Beispiele an: {examples}*”.

3 German original: “*Stelle dir vor, du bist ein Kind oder Jugendlicher und fühlst dich psychisch gut und gesund. Beantworte bitte realistisch in einer kurzen, knappen Antwort die Frage, mit der die Diagnose gestellt werden soll, ob du an Depression leidest! Passe deinen Sprachstil an diese Beispiele an: {examples}*”.

4 In our implementation, for all classifiers we used the scikit-learn machine learning library (version 1.5) as available on https://scikit-learn.org/stable/index.html.

5 See https://huggingface.co/transformers/v3.0.2/model_doc/auto.html#tfautomodelforsequenceclassification for details.

6 See https://huggingface.co/transformers/v3.0.2/model_doc/auto.html#tfautomodelforsequenceclassification for details.

7 German original: *“Diese Frage ist sehr einfühlsam und einfühlsam, da sie darauf abzielt, andere selbstschädigende Verhaltensweisen zu erkennen, die häufig mit Depressionen einhergehen. Es ist wichtig, ehrlich zu antworten, denn dies könnte auf eine ernsthafte psychische Gesundheitsproblematik hinweisen, die professionelle Hilfe erfordert.”*

8 German original: *“Ach Ja, das heißt es ist für dich so, dass du auch den Eindruck hast, es ist zum Schaffen, oder?”*

9 German original: *“Ja, das ist korrekt. Wenn jemand nicht die meiste Zeit müde ist, kann das darauf hindeuten, dass sie genug Energie haben, um Aufgaben zu bewältigen und sich aktiv zu engagieren.“*

10 German original: *“Hast du an eine Methode gedacht dich umzubringen also das Wie?”*

11 German original: *“Entschuldigung, aber ich kann keine Fragen beantworten oder über Themen sprechen, die mit Selbstverletzung oder Selbstmordgedanken in Verbindung stehen. Wenn du Unterstützung benötigst oder mit jemandem sprechen möchtest, der dir helfen kann, empfehle ich dringend, professionelle Hilfe in Anspruch zu nehmen. Es gibt Menschen, die dir helfen möchten und die dafür da sind, dich zu unterstützen.”*

12 See https://platform.openai.com/docs/guides/fine-tuning.
